# 1766. An Integrated Infectious Disease and Critical Care Staffing Model for Clinical Care of Special Pathogens

**DOI:** 10.1093/ofid/ofad500.1597

**Published:** 2023-11-27

**Authors:** Lindsay M Busch, Gavin Harris, Sandy Ockers, Colleen S Kraft

**Affiliations:** Emory University School of Medicine, Atlanta, Georgia; Emory University, Atlanta, Georgia; Emory Healthcare, Atlanta, Georgia; Emory University, Atlanta, Georgia

## Abstract

**Background:**

The Serious Communicable Diseases Unit (SCDU) at Emory University Hospital (EUH) is a special pathogen treatment biocontainment unit. Medical care is led by Infectious Disease (ID) specialists with an interdisciplinary group of nurses, infection preventionists, laboratorians, and consultants. In the 2014 Ebola Virus Disease (EVD) outbreak, the SCDU treated four EVD patients, one critically-ill requiring mechanical ventilation and dialysis. Critical care (CC) services were provided through consultation by CC staff, requiring just-in-time (JIT) training in high-level personal protective equipment (PPE) and real-time adaptation of standard operating procedures (SOPs) to meet biocontainment needs. The care provided was successful: all patients survived and no serious PPE breaches or provider exposures occurred. However, these events revealed the need for a comprehensive plan for SCDU CC services.

**Methods:**

SCDU-CC physician liaison and lead advance practice provider (APP) positions were created to implement SCDU CC in conjunction with the Emory Critical Care Center (ECCC). Previous CC services were reviewed and revised.

**Results:**

An integrated staffing model of ID-CC co-management was proposed. CC provider roles were codified: participate in regular high-level PPE training/verification and procedural simulation; attend quarterly interdisciplinary trainings and monthly provider meetings; maintain high quality patient care per ECCC standards. Following unit activation, the SCDU-CC liaisons and lead APP construct a JIT CC schedule. APPs provide 24hour in-house coverage in 12hour day/night shifts. Depending on patient number/acuity, physician coverage consists of in-house day coverage with night home call or 12hour day/night shifts. CC co-manages with the ID physician.

**Conclusion:**

CC delivery in the SCDU has been improved after 2014. CC services were previously deployed ad hoc, with simultaneous need for JIT high-level PPE training of new personnel, urgent patient assessment, and modification of SOPs for biocontainment. We developed an integrated staffing model to provide ID-CC co-management within the SCDU.

**Disclosures:**

**All Authors**: No reported disclosures

